# Pershore Cottage Hospital

**Published:** 1896-07-18

**Authors:** 


### EDITOR'S LETTER-BOX.

[Our correspondents are reminded that proxility is a great bar to publication, and that brevity of style and conciseness of statement greatly facilitate early insertion.]

### PERSHORE COTTAGE HOSPITAL.

MR. J. H. WILLIAMS, architect of the above institution, writes: My attention has been called to your notice of the Pershore Cottage Hospital, and it appears that instead of using the information I supplied you with fairly you have gone out of your way to try and injure my positon. I will take your report *seriatim*, beginning with (1) "This passage derives some light, &c., and would be dark, &c." You give a pious opinion based on theory and without knowing anything of the size, &c., of staircase window, and you are altogether wrong. It is not dark, but, on the contrary, well lighted. (2) "The kitchen and scullery are on opposite sides of this passage, &c.," but were purposely so placed so that in summer time most of the kitchen work would be done in the scullery, keeping the kitchen as a more comfortable sitting-room for the servants. (3) Moveable baths were specially asked for by the committee, who also approved their disposition, not needing bath-room separately. (4) "Duty room" for nurses and many other things were thought of—you should not assume they were not because they are not included in the plan, they were only omitted for want of money—and the committee expected to get more accommodation than first provided for some £200 less than was expended.

(5) The verandah does not make the kitchen dark (so those say who use it), but affords a pleasant screen from a strong midday sun and a shelter for convalescents in the evening. (6) Each ward is for two beds, which are well placed in the one case, and when the other ward is built will be equally so there. I was careful in my letter to say that "I had added a little to the plan showing the ultimate arrangement of w.c.'s, &c." From this, I think, you should have inferred that the others now provided would be for nurses' accommodation only, but you ignore this fact in your report altogether. Then again when the whole scheme is complete the ward on the right would be dominated from the nurses' sitting-room. Your remarks savour very much of a personal attack.

[The writer of the notice assures us that there was not the slighest intention of hurting Mr. Williams's feelings in preparing the report, still less of injuring his reputation, and, so far as his letter explains omissions and refutes criticisms, it is worthy of all publicity. The suggestion of "a personal attack" is, however, absurd. Except as the author of the published plans, Mr. Williams's name is absolutely unknown to the writer of the report. (1) The direct light from the staircase window to the passage is blocked by the angle of the w.c. wall. The lighting of a long passage in any domestic building only by what filters down the wall of a staircase is to be deprecated; in hospital buildings this is especially undesirable; every portion of such buildings should have a separate and ample supply of light and air. (2) The fact that the scullery and kitchen were purposely placed on opposite sides of a principal passage does not alter the obvious inconvenience and disagreeable consequences of such an arrangement. To plan a kitchen with a sunny aspect is a mistake in first principles. (3) No suggestion of a bath or space for a bath was shown on the plans; the obvious inference being that none was contemplated. (4) It seems a pity to complete plans and buildings for hospital purposes without so essential a provision as a duty-room and space for appliances necessary to nursing work. It is good they were thought of, bad that they were omitted. (5) A kitchen should have a cool aspect, and enjoy all the light it can, but should not be exposed to the rays of "the mid-day sun" in any case. (6) A reference to the plan makes it difficult to see how two beds can be placed in either ward clear of the walls, and also clear of all cross-draught between the windows, door, and fireplace; and a patient's bed should not be exposed to such draughts. (7) The letter suggesting an ultimate and better arrangement of w.c.'s did not reach the writer of the report; and the provision presented on the plans seems very inadequate. (8) Each ward in every cottage hospital should be controlled by a nurse. If there is only one in charge, as seems probable in this case, then her room should command the wards.—Ed. *T. H.*]

